# Analysis of microbial diversity and succession during *Xiaoqu Baijiu* fermentation using high‐throughput sequencing technology

**DOI:** 10.1002/elsc.202200015

**Published:** 2022-06-14

**Authors:** Qing Wang, ChaoYan Wang, XiaoQing Xiang, HaiLin Xu, GuoQiang Han

**Affiliations:** ^1^ School of Advanced Agriculture and Bioengineering Yangtze Normal University Chongqing People's Republic of China

**Keywords:** *Baijiu*, high‐throughput sequencing, microbial diversity, microbial succession, Xiaoqu

## Abstract

In this study, high‐throughput sequencing (HTS) was used to compare and analyze the microbial diversity and succession during the brewing process of *xiaoqu Baijiu*. A total of 34 phyla and 378 genera of bacteria, as well as four phyla, 32 genera of fungi were detected. At the phylum level, Firmicutes, Proteobacteria, Ascomycota, and Mucoromycota were the dominant groups. During the brewing process of *xiaoqu Baijiu*, the dominant bacteria were *Weissella* and unidentified *Rickettsiales* within the first 2 days of brewing, followed by *Lactobacillus* at 3 days until to the end of brewing. The dominant fungi were *Rhizopus*, *Saccharomyces*, and *Issatchenkia*. The relative abundance of *Rhizopus* decreased with the extension of brewing time, while the relative abundance of *Saccharomyces* increased, and *Saccharomyces* became the dominant species at the second day of brewing. This study revealed the diversity and changes of the microbial community during the brewing process of *xiaoqu Baijiu*, providing theoretical support and laying a foundation for future study on the contribution of microbial metabolism during brewing of *xiaoqu Baijiu*, thereby promoting the development of *xiaoqu Baijiu* industry.

**Abbreviations**
HTS: high‐throughput sequencing
ITS1: internal transcribed spacer1
LABs: lactic acid bacteria
OTUs: operational taxonomic units
PCA: principal component analysis
*R. oryzae*
: *Rhizopus oryzae*
*S. cerevisiae*
: *Saccharomyces cerevisiae*

## 1 INTRODUCTION

Chinese liquor (*Baijiu*) is one of the oldest solid‐state fermented, distilled spirits [1]. There are some challenges during traditional solid‐state *Baijiu* brewing process [2]. It is difficult to monitor fermentation parameters because the containers are usually set underground. Meanwhile, the parameters (such as temperature, pH) are often not uniform. In addition, it is also difficult to apply modern automatic productin lines to the production of *Baijiu*, because fermentaion, distillation and storage are batch operations during the production of *Baijiu* [2].

According to the aroma components, *Baijiu* can be categorized into three major types, sauce‐flavor *Baijiu*, strong‐flavor *Baijiu*, and light‐flavor *Baijiu* [3]. *Xiaoqu Baijiu* is an important type of light‐flavor *Baijiu* in China, which is produced using *xiaoqu*, one of the major types of *jiuqu* starter used in *Baijiu* production*. Xiaoqu* is usually made from rice, rice bran and wheat bran as the raw material, which is artificially inoculated with *Rhizopus* and yeast [4, 5]. *Xiaoqu Baijiu* had a short brewing period and a higher yield compared with *Daqu Baijiu* [5].

In early studies, microbial diversity was mostly investigated using traditional microbial culture methods. With the rapid development of molecular biotechnology in recent years, polymerase chain reaction denaturing gradient gel electrophoresis (PCR‐DGGE) and high‐throughput sequencing (HTS) technology have become the standard approaches for the analysis of microbial community diversity [6]. HTS is a simple technique that generates large amount of data [7]. HTS technology has been increasingly applied to study the diversity and structure of the microbial community during the *Daqu Baijiu* brewing process, including *Daqu*, the mud used to seal the fermentation pit, and fermented grains [8, 9, 10, 11]. Microorganisms produce a majority of flavored substances during the brewing of *Baijiu* [12]. Therefore, there is an increasing interest in revealing the characteristics of the microbial community and the important influence of its changes on *Baijiu* brewing [13]. Bacterial flora was found to significantly influence the production and quality of sauce‐flavored and strong‐flavored *Baijiu* [7, 14]. However, few studies investigated the microbial diversity and community structure of light‐flavor *Baijiu* [15], especially *xiaoqu Baijiu*. Studies of the microbial diversity in *xiaoqu Baijiu* mainly focused on *xiaoqu* from different production areas in China, which possess different bacterial compositions [6, 16, 17]. Physicochemical indexes and different metabolites of *xiaoqu Baijiu*, such as metabolism of carbohydrates, cofactors, vitamins, and amino acids, were closely correlated with microbiota of *xiaoqu* [18, 19]. *Lactobacillus*, *Bacillus*, *Acinetobacter* and *Gluconobacter* were dominant bacteria in x*iaoqu* from Hubei and Sichuan provinces of China [20]. *Lactobacillus*, *Leuconostoc* and *Saccharomycopsis*, *Rhizopus*, *Aspergillus* were the most abundantly present bacterial and fungal genera in *Zangqu* [21]. However, there is little research focused on the microbial community structure and diversity during the *xiaoqu Baijiu* brewing process. The abundance of *Bacillus* was the highest in the early stage of fermentation, while abundance of the lactic acid bacteria (LABs) increased to the highest with the progress of fermentation [22]. In this study, the structure and diversity of fungi were not investigated. *Klebsiella*, *Saccharomycetales*, and *Rhizopus oryzae* were dominated in Sichuan *xiaoqu Baijiu* fermented grains [23]. The result was different from the results reported before [22], which maybe due to the region or the variety of *xiaoqu* used. The numbers of yeasts and bacteria were significantly higher in new mechanical technology than that in traditional technology at the peak of *xiaoqu Baijiu* fermentation [24]. The results indicated that brewing technology also influenced the microbial diversity and succession.

In the present study, we investigated the changes of microbial community structure and diversity during *xiaoqu Baijiu* brewing. It is helpful to understand the contribution of various microorganisms to the quality of *xiaoqu Baijiu*. Pure cultured *xiaoqu* was used to better understand the source and role of functional bacteria in *xiaoqu Baijiu* by deep mining of functional taxa. Understanding the microbial community structure and microbial succession during *xiaoqu Baijiu* brewing will provide a scientific basis for quality control and flavor enhancement of *xiaoqu Baijiu*.

**PRACTICAL APPLICATION**

Microbial diversity and succession have significantly influence on the production and quality of *Baijiu*. This research studied the microbial diversity and succession during *Xiaoqu Baijiu* fermentation. The results show that *Weissella*, *Lactobacillus*, *Rhizopus*, *Saccharomyces*, and *Issatchenkia* were important for *xiaoqu Baijiu* brewing. *Rhizopus* and *Saccharomyces* were provided by *xiaoqu* while *Lactobacillus* come from fermentation environment. Therefore, improvement of strains in *xiaoqu* and fermentation environment can change microbial diversity during *xiaoqu Baijiu* brewing process, and then improve the quality of *xiaoqu Baijiu*. This study provides new insights in depth studies of the microbial diversity and quality improvement of *xiaoqu Baijiu*.

## 2 MATERIALS AND METHODS

### 2.1 Sample preparation and collection

Samples were collected from the distillery of Wangxian Co. Ltd., Chongqing, China. The pure culture *xiaoqu* was prepared by inoculating wheat bran with a 7:2:3:1 mixture of *Aspergillus niger*, *Rhizopus nigrum*, *R. oryzae*, and *Aspergillus oryzae* at a ratio of 0.4% of the weight of raw materials. The aroma‐producing *S. cerevisiae* was added according to 0.5% of the weight.

**FIGURE 1:**
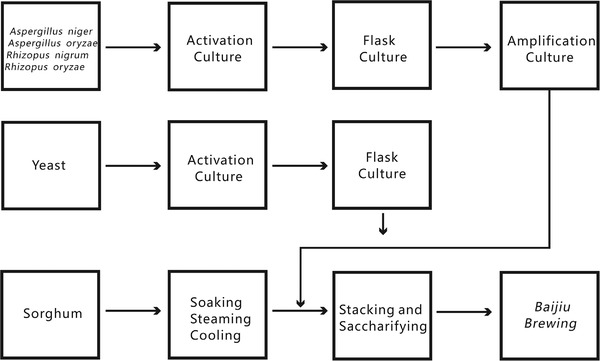
The brewing process of *xiaoqu Baijiu*

After soaking, steaming, cooling, mixing with *xiaoqu*, stacking and saccharification for 24 h, sorghum was put into the pit and sealed with mud. The pit was made of cement, and the brewing cycle was 7 days (Figure 1). The microbial species and abundance changed greatly in the early stage of brewing, and tended to stabilize in the later stage. Therefore, the samples were taken at the end of the stacking brewing stage (Day 0), everyday during the first three days (Day 1, Day 2, Day 3), and on the fifth and last day (Day 5, Day 7) in the *Baijiu* brewing stage. The samples were taken at 30 cm below the surface of fermented grains. The samples were frozen in liquid nitrogen immediately after collection, and then stored at ‐80°C for subsequent DNA extraction.

### 2.2 Determination of pH, temperature, moisture content, and ethanol concentration

Samples of grains were stirred in distilled water for 30 min and then pH were measured with a pH meter (Mettler Toledo Co., Ltd. Switzerland). Temperature was monitored by an electronic thermometer during fermentation. Samples of grains were baked in an oven until a constant weight reached and measured for moisture content. Samples of grains and water were distilled for determination of alcohol content [23].

### 2.3 DNA extraction and HTS

Genomic DNA of the samples was extracted using the CTAB method [22]. The purity and concentration of the DNA were assessed by agarose gel electrophoresis. A suitable DNA sample was diluted with nuclease‐free water to 1 ng/ L.

The V4 hypervariable region of bacterial 16S rDNA was amplified using the primer pair 515F/806R (515F: GTGYCAGCMCCGCGGTAA, 806R: GGACTACNVGGGTWTCTAA). The fungal ITS1 hypervariable region was amplified using the primer pair 1737F/2043R (1737F: GGAAGTAAAGCTGTAACAGG, 2043R: GCTGCGTTCTTCATCATCGATGC).

All PCR products were purified and used for library construction using the TruSeq® DNA PCR‐Free Sample Preparation Kit. After the library was qualified by qubit and Q‐PCR, samples were loaded onto the Illumina NovaSeq6000 platform (Novogene Co., Ltd. China) for HTS. The raw data has been uploaded to NCBI SRA (BioProject ID: PRJNA687145).

### 2.4 Data analysis

The raw HTS data, with reads spliced using FLASH software [25], was processed using QIIME [26]. Final effective data was obtained through tag interception and length filtering, and then clustered into operational taxonomic units (OTUs) with 97% sequence identity using VSEARCH software [27, 28]. The OTU sequences were annotated via database comparisons using Mothur, and the relative content of OTUs in each sample was calculated [29]. Shannon diversity and Chao1 richness were calculated on the basis of the OTUs [30]. A map of bacterial community composition was drawn using R software (R Foundation for Statistical Computing, Vienna, Austria).

## 3 RESULTS

### 3.1 Physicochemical indicators of fermented grains

The pH tended to decrease during fermentaion (Table 1). The temperature first increased from 22 to 35°C from day 0 to day 3 and maintained to day 5, then decreased to 31°C at day 7. The water content increased rapidly from day 0 to day 3. The ethanol content first increased rapidly before day 5 and then increased slowly till to the end of fermentation.

### 3.2 Microbial abundance and OTUs diversity

In order to study the species composition of each sample, sequences with 97% identity were clustered into OTUs and annotated. A total of 1609 OTUs based on the bacterial 16S rRNA V4 region and 57 OTUs based on the fungal ITS1 region were obtained by clustering. A total of 34 phyla, 378 genera and 215 species of bacteria, as well as four phyla, 32 genera, and 44 species of fungi were annotated after searching each OTU against the taxonomic database.

**FIGURE 2:**
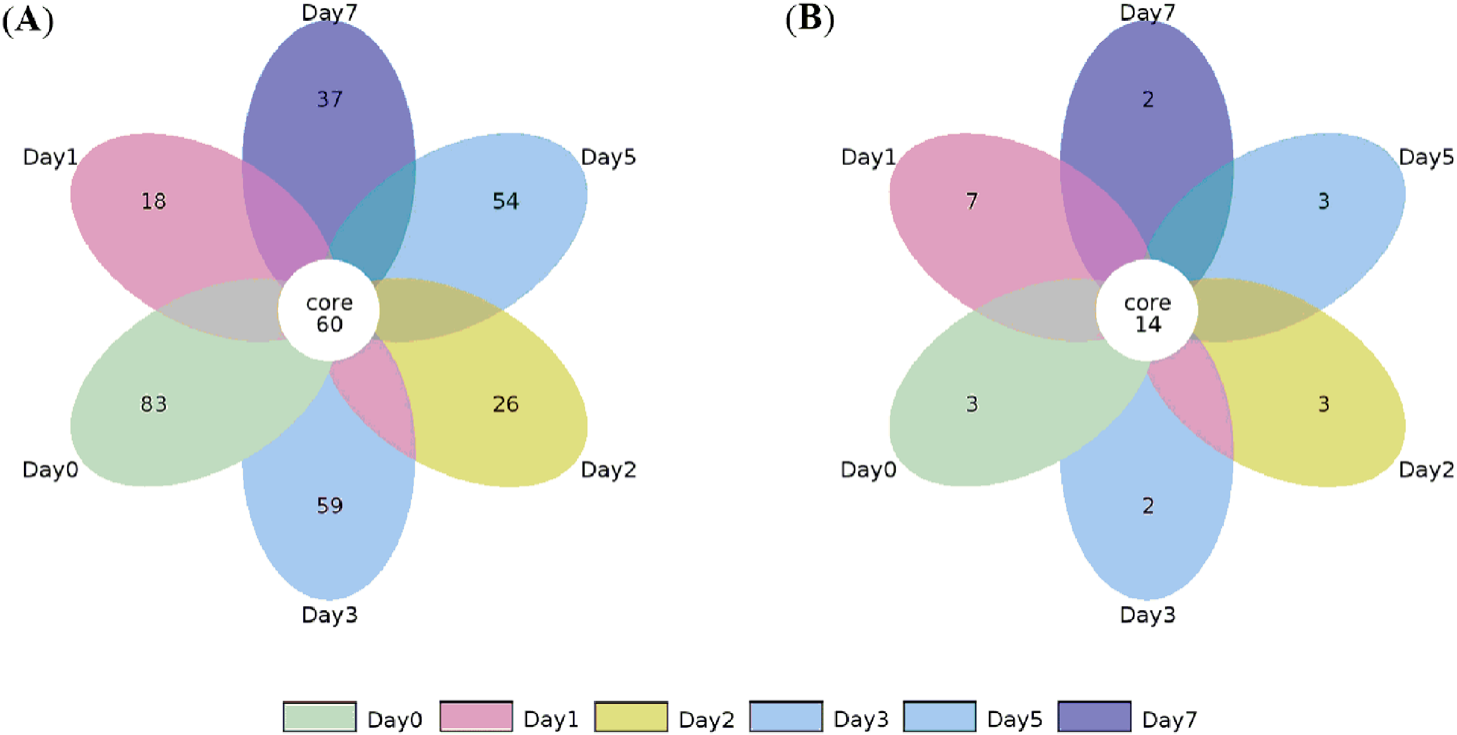
Statistics and comparison of OTUs in samples from different stages of the *xiaoqu Baijiu* brewing process. (A) OTUs of bacteria; (B) OTUs of fungi

The OTUs of bacteria in all samples from different stages of the *xiaoqu Baijiu* brewing process were quantified and compared. A Venn diagram was created to investigate potential exclusively shared OTUs. As shown in Figure 2, at a 97% similarity level, 60 bacterial and 14 fungal OTUs were found in all of the six samples. The results suggested that the fungal community is less complex than the bacterial community in the brewing process of *xiaoqu Baijiu*. The number of unique OTUs decreased during brewing.

The community abundance and diversity indexes, including Shannon, Simpson, ACE and Chao1, were calculated to assess the abundance and diversity of microbial OTUs (Table 2). The Chao1 and Shannon indexes of each sample were used to evaluate the richness and diversity describe the alpha‐diversity of the microbial community, respectively. The Chao1 index was positively correlated with the microbial community abundance. The average coverage of all samples exceeded 0.99, indicating that the identified sequences were representative of the actual community in the sample. The ACE and Chao1 values decreased on the first day of brewing, and then gradually increased, reaching the highest value on the fifth day of brewing. The Shannon and Simpson indexes were used to estimate the microbial diversity of the samples. Both indexes reached the highest values at the second day of brewing, while bacterial diversity was highest on day 7 in the study reported [23]. The Shannon diversity of traditional xiaoqu group ranged from 2.27 to 3.52, higher than the results of Dong et al. [22]. Higher Shannon index values indicate greater microbial diversity. This indicates that microbial diversity was highest at the second day of brewing in this study. Generally, the abundance and diversity of bacteria in the *xiaoqu Baijiu* samples decreased with prolonged brewing time.

For fungi, the ACE index increased at the first day, and decreased on the second day. The Chao1 reflects the community richness, and its highest value of 38.6 was observed on the first day of brewing. The Shannon index was lowest at the beginning of brewing and reached the maximum at the third day of brewing, while fungal diversity was highest on day 1 and lowest on day 7 in the study reported before [23]. The Simpson index can also be used to estimate the microbial diversity of the sample, and it was also the lowest at the beginning of brewing (Table 2).

**TABLE 1:** The pH, temperature, moisture content and ethanol concentration of samples from different stages of the *xiaoqu Baijiu* brewing process

**Samples**	**pH**	**Temperature**	**Moisture content (%)**	**Ethanol (%)**
Day 0	5.84 ± 0.21	22.45 ± 0.23	56.31 ± 1.83	–
Day 1	5.43 ± 0.15	26.76 ± 0.83	61.82 ± 2.14	0.52 ± 0.11
Day 2	5.08 ± 0.13	30.47 ± 1.26	67.57 ± 1.73	2.97 ± 0.17
Day 3	4.53 ± 0.19	34.17 ± 1.48	68.92 ± 1.91	4.64 ± 0.26
Day 5	4.13 ± 0.11	34.65 ± 0.92	71.35 ± 2.32	5.11 ± 0.24
Day 7	4.07 ± 0.12	31.36 ± 1.13	69.18 ± 2.17	5.35 ± 0.18

*Note*: Each value represents the mean of three determination ± standard deviations. (–) not detected.

**TABLE 2:** Richness and diversity indexes of the microbial community in samples from different stages of the *xiaoqu Baijiu* brewing process

	**Bacterial**				**Fungi**			
**Samples**	**Shannon**	**Simpson**	**Chao1**	**Ace**	**Shannon**	**Simpson**	**Chao1**	**Ace**
Day 0	2.95	0.81	179	183	0.75	0.21	28	30.22
Day 1	2.33	0.64	134	135	1.57	0.57	39	42.79
Day 2	3.52	0.81	155	159	1.71	0.65	28	31.70
Day 3	2.27	0.59	146	150	1.77	0.66	26	27.00
Day 5	2.79	0.77	171	176	1.62	0.65	28	32.34
Day 7	3.06	0.77	145	147	1.43	0.56	28	33.20

### 3.3 Diversity analysis of the bacterial community

**FIGURE 3:**
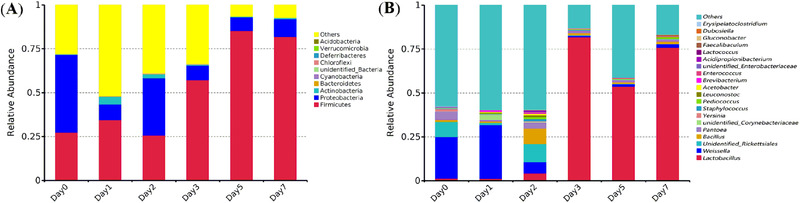
Bacterial community structure of samples at phylum level and genus level. (A) at phylum level; (B) at genus level

At the phylum level, 34 bacterial species were identified in the samples. The top 10 species in abundance and related species are shown in Figure 3. The dominant bacterial phyla was Proteobacteria and Firmicutes (Figure 3A). In the early stage of brewing, the dominant bacteria were Proteobacteria. The proportion of Proteobacteria decreased during the brewing of *xiaoqu Baijiu*, and Firmicutes became the dominant group, which may be because some aerobic Proteobacteria did not survive in the environment of low oxygen, high acid and alcohol. There was still a certain relative abundance of Actinobacteria during the first 2 days brewing, but their abundance was reduced to a very low level with prolonged brewing time.

A total of 378 bacterial species were detected in *xiaoqu Baijiu* samples at the genus level. The top 20 most abundant genera and related species merged are shown in Figure 3B. Although the dominant bacterial genera (top 20) were almost identical, there were significant differences in bacterial abundance among the samples. The top five genera were *Lactobacillus*, *Weissella*, unidentified *Rickettsiales*, *Bacillus*, and *Pantoea*. The relative abundance of *Weissella* and unidentified *Rickettsiales* accounted for a large proportion in the early stage of brewing and decreased during brewing. The relative abundance of *Bacillus* increased before the second day of brewing. The relative abundance of *Pantoea* did not change much during brewing process. The relative abundance of *Lactobacillus* did not change much in the first 2 days of brewing, but increased rapidly during the brewing process, and became the dominant bacteria in the late stage of brewing.

### 3.4 Diversity analysis of the fungal community

**FIGURE 4:**
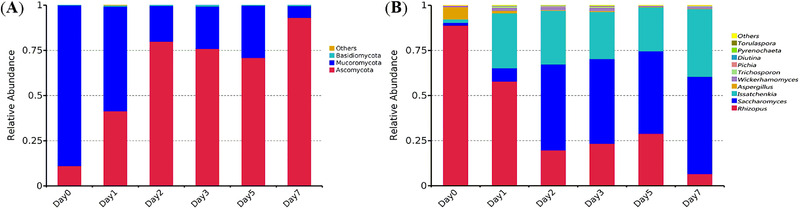
Fungal community structure of samples at phylum level and genus level. (A) at phylum level; (B) at genus level

At the phylum level, four fungal species were identified in the samples. As shown in Figure 4, the dominant fungi were Ascomycota and Mucoromycota at the phylum level. During the brewing process, the starch content of the substrate progressively decreases, and the relative abundance of Mucoromycota decreased. The community of Ascomycota increased and occupied a dominant position during the brewing process, which indicated that Ascomycota play an important role in the brewing of *xiaoqu Baijiu*.

At the genus level, a total of 32 fungal species were detected in the *xiaoqu Baijiu* samples. The top 10 genera and related species are shown in Figure 4B. *Rhizopus* and *Aspergillus* were the dominant genera before the material paced into the fermentation pit, but their relative abundance decreased during brewing. The relative abundance of *Saccharomyces* increased during the entire brewing process and it became the dominant genus at the end of brewing. The relative abundance of *Issatchenkia* increased at the beginning of the brewing process, but did not change much during late stage of the brewing.

### 3.5 Sample clustering in bacterial and fungal communities

**FIGURE 5:**
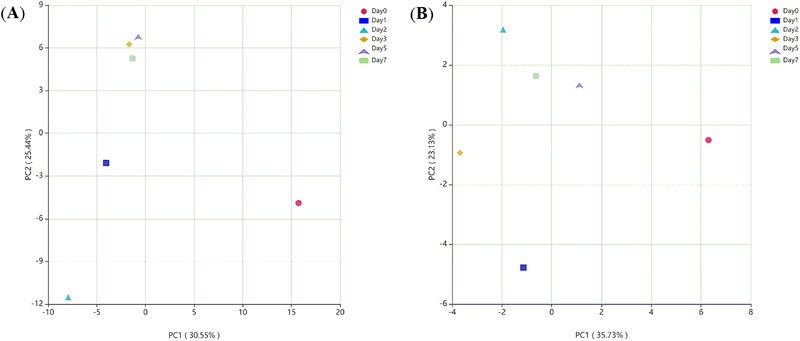
Principal component analysis (PCA) of bacteria and fungi at the genus level. (A) PCA of bacteria; (B) PCA of fungi

Principal component analysis (PCA) was used to analyze the changes of relative abundance and diversity of the microbial community on a two‐dimensional coordinate chart. The variation in the bacterial communities between samples was found to be 30.55% (PC1) and 25.44% (PC2) with a strong separation by region as shown in Figure 5A. Samples Day 3, Day 5, and Day 7 were close together, indicating that the bacterial composition are similar and more stable. Samples Day 0, Day 1, and Day 2 were far from these, indicating that fermentation process had impact on the bacteria of samples. For the fungal community, the maximal variation values were 35.73% (PC1) and 23.13% (PC2) as shown in Figure 5B. Samples were scattered, and their fungal composition were relatively different, indicating there were some differences in fungal diversity during *Baijiu* brewing stages.

## 4 DISCUSSION

This study applied HTS to reveal the microbial diversity and succession during x*iaoqu Baijiu* fermentation. The bacterial dynacmics were similar with the report of Dong et al. [22]. The increase of relative abundance of LAB was mainly due to *Lactobacillus* in the late stage of fermentation. However, *Weissella* was the predominant LAB in the early stage of *xiaoqu Baijiu* brewing in our study while *Lactococcus* was the predominant LAB in the report of Dong et al. [22]. *Bacillus* exhibited higher abundance only at the second day of fermentation in our study, which was also different with reported before. *Klebsiella* was not found in top 20 most abundant genera (Figure 3B), while it is the dominant microorganism during brewing of Sichuan *Xiaoqu Baijiu* [23]. *Saccharomyces* and *Issatchenkia* were the dominant fungal in the late stage of fermentation (Figure 4B), while *Pichia kudriavzevii* was more active than *S. cerevisiae* in the study of Hu et al. [24]. Different microbial diversities during *xiaoqu Baijiu* brewing process might be attributed to three factors, *Xiaoqu*, fermentation conditions and environment. There are similarities and differences between our study and previous studies, which provide guidance for *xiaoqu Baijiu* brewing. The improvemet of *Xiaoqu*, optimization of fermentation conditions and environment are directions to improve the quality of *xiaoqu Baijiu*.

*Bacillus* in the starter can secret various hydrolases, including amylases, proteases, and lipases, which hydrolyze macromolecular substrates and produce specific flavor compounds such as acetate, higher alcohols, diacetyl, pyrazines and aromatic compounds, improving the sensory experience and conferring health benefits [31]. Moreover, a synergistic interaction between *Bacillus* and *Aspergillus* leads to a richer spectrum of aromatic compounds [11]. Addition of *Bacillus* in *xiaoqu* provide a way to improve the flavor of *xiaoqu Baijiu*. *Pantoea* is an important heterotrophic bacterium that is often found in brewing. Although the abundance of *Acetobacter* and *Gluconobacter* was not high during the brewing process, they can produce acetic acid as a precursor of ethyl acetate, which is a major flavor component of *xiaoqu Baijiu* [23].

*Lactobacillus, Weissella*, *Pediococcus*, *Leuconostoc*, and *Lactococcus* are LABs. The increase trend of Firmicutes in the process of *Baijiu* brewing was mainly related to the increase in abundance of LABs [31,^32^]. As facultatively anaerobic or aerotolerant bacteria [33], LABs have an important role in regulating the bacterial community during *Baijiu* brewing. LABs also predominate during the brewing stage of sauce‐flavored, strong‐flavored, and light‐flavored *Baijiu* [7, 14, 32]. Lactic acid and acetic acid, produced by LABs, can react with ethanol to produce ethyl lactate and ethyl acetate, which are important flavor components of *xiaoqu Baijiu* [30]. However, too much ethyl lactate causes an undesirable aroma in light‐flavored *Baijiu*. Lactic acid, produced with the increase of LABs, reduces the pH of the fermented grains, thereby greatly influencing the community structure [30] and inhibiting the propagation of spoilage bacteria [34]. A negative correlation between LABs and other prokaryotic groups was observed during the brewing process [30]. LABs also possess different enzymes such as glucosidases, esterases and proteases, which play a critical role in the formation of volatile compounds in fermented foods [35, 36]. *Lactobacillus* significantly enhanced the generation of 3‐(methylthio)‐1‐propanol and dimethyl disulfide when cocultured with *S. cerevisiae*, though it could not produce these two compounds. In addition, the related gene transcriptions in *S. cerevisiae* and *Lactobacillus* were significantly enhanced [37]. The type, quantity and dynamic change of LABs species are crucial determinants of the flavor of *Baijiu*. LABs may be introduced from the environment of the *xiaoqu Baijiu* brewing workshop during sorghum cooling, because no LABs are added to *xiaoqu*. In a previous study, the abundance of LABs on the surface of the ground and tools in the brewing workshop was indeed found to be very high [38].

Molds and yeasts are mainly responsible for saccharification and alcoholic brewing during the principal production of *Baijiu* [39]. *Rhizopus* and *Aspergillus* are important functional microorganisms in many fermented foods [16]. The relative abundance of *Aspergillus* and *Rhizopus* decreased due to the continuous increase of the alcohol and acid concentrations, as well as the lack of oxygen. *R. oryzae* has a high amylase production capacity, and is often used to hydrolyze starch from sorghum in *koji* and *Baijiu* brewing [40]. *Rhizopus* also affects *Baijiu* flavor by producing glycerol, lactic acid, enzymes and volatile compounds, such as ethanol, 2‐methyl‐1‐butanol, and 3‐methyl‐1‐butanol [41]. *Aspergillus* produce a wide spectrum of proteolytic and other hydrolytic enzymes, playing an important role in the saccharification of starch and protein hydrolysis in sorghum [42]. *Aspergillus* can also promote the synthesis of certain esters, improving the flavor of *Baijiu*. This is the reason why *Rhizopus* and *Aspergillus* were added in *xiaoqu*.

With its strong ethanol brewing capacity under anaerobic conditions, *Saccharomyces* is responsible not only for the alcohol yield but also for the flavor and taste features of *xiaoqu Baijiu* [16,^43^]. *Saccharomyces* also interacts with other microorganisms such as molds and bacteria, improving the quality of *xiaoqu Baijiu*. Since *Saccharomyces* can tolerate anaerobic conditions and high ethanol concentrations, it is unsurprising that its relative abundance increased during the *xiaoqu Baijiu* brewing process. *Issatchenkia*, a yeast that is abundantly found in soil and fermented grains, can produce ethanol and ethyl acetate, and resistant to acid, high temperatures and ethanol. It is a functional species in the brewing process of *Baijiu*, and was found in many samples of *Daqu* and fermented grains used for the production of *Baijiu* with different flavor types [38, 44].

*Baijiu* can maintain its character due to the similar microbial communities of different batches in the same period. The difference of microbial communities in different workshops or periods may cause the different flavor profiles even if the exact same processing methods were used [13]. The abundance and diversity of microorganisms such as bacteria, yeasts and molds in fermentation starters, more conducive to the production of flavor compounds, is crucial for the production of high‐quality *Baijiu* [45]. These factors are influenced by changes of the microenvironment, such as reduction of substrate, decrease of pH, increase of alcohol concentration, anaerobic conditions, as well as biotic and abiotic interactions [46, 47].

Because *xiaoqu* and *xiaoqu Baijiu* are produced in an open environment, the contained microorganisms are derived not only from the starters, but also from the environment encountered in the process of material pretreatment, including the water, machines, tools, ground and air in the material pretreatment room [4,^48^]. Consequently, the quality of *Baijiu* depends on a well‐balanced microbial community derived from both the production environment and the starter. Microorganisms such as *Lactobacillus*, *Bacillus*, and *Saccharomyces* are thought to be introduced into sorghum from the brewing workshop environment during cooling and enter the alcoholic brewing stage [38]. *Lactobacillus* and *Saccharomyces* were the predominant bacterial and fungal genera at the later stages of brewing, and they are closely related to the formation of various flavors during *xiaoqu Baijiu* brewing.

A more comprehensive understanding of the microbial diversity and microbial succession during the brewing process of *xiaoqu Baijiu* was obtained. The microbial community structure and diversity showed a microbial succession during the *xiaoqu Baijiu* brewing process. This study expands our understanding of the microbial community diversity of *xiaoqu Baijiu* during the brewing process and provides useful information for future in depth studies of the microbial diversity and quality improvement of *xiaoqu Baijiu*.

## CONFICT OF INTEREST

The authors declare that they have no conflicts of interest related to the publication of this paper.

## Data Availability

All data generated or analyzed in this study are included in this paper. The datasets can be found in online repositories. The names of the repositories and accession number can be found at: https://www.ncbi.nlm.nih.gov/, PRJNA687145.
